# Case Report: Potocki-Lupski Syndrome in Five Siblings

**DOI:** 10.3389/fped.2021.698629

**Published:** 2021-11-08

**Authors:** Alina Grama, Claudia Sîrbe, Diana Miclea, Simona Sorana Cǎinap, Delia Huniadi, Bogdan Bulata, Tudor Lucian Pop

**Affiliations:** ^1^Second Pediatric Discipline, Department of Mother and Child, University of Medicine and Pharmacy Iuliu Hațieganu, Cluj-Napoca, Romania; ^2^Second Pediatric Clinic, Emergency Clinical Hospital for Children, Cluj-Napoca, Romania; ^3^Discipline of Medical Genetics, Department of Molecular Sciences, University of Medicine and Pharmacy Iuliu Hațieganu, Cluj-Napoca, Romania; ^4^Discipline of Neurology, University of Medicine and Pharmacy Iuliu Hațieganu, Cluj-Napoca, Romania; ^5^Pediatric Nephrology, Dialysis and Toxicology Clinic, Emergency Clinical Hospital for Children, Cluj-Napoca, Romania

**Keywords:** children, craniofacial dysmorphism, hypotonia, cognitive delay, 17p112 duplication, Potocki-Lupski syndrome

## Abstract

Potocki-Lupski syndrome (PTLS) is a rare developmental disorder resulting from the partial duplication of the short arm of chromosome 17. Affected children may have hypotonia, facial dysmorphism, or neurological abnormalities. PTLS is also frequently associated with failure to thrive due to swallowing difficulties or growth hormone deficiency. We report the first Romanian family (a mother and her five children) diagnosed with PTLS (17p11.2 microduplication). Fortunately, they present a less severe form of the disease. The neurological manifestations (speech delay, mild intellectual disability) are associated with craniofacial dysmorphism (microcephaly, micrognathia, triangular face, broad forehead, long chin, prominent ears, dolichocephaly, down slanting palpebral fissures). The diagnostic was established using a multiplex ligation-dependent probe amplification technique (MLPA) test, which detected the duplication of three regions of the 17p11.2 chromosome (RAI1, DRC3-6, LLGL1-4RA). Children with PTLS have specific phenotypes (craniofacial dysmorphism or neurological manifestations), which must draw the pediatrician's attention to a possible genetic condition. However, every child with this disease is unique and may have a different clinical presentation. A multi-disciplinary team is needed for the management of these patients. The parent's counseling and genetic advice are essential for a family with children with PTLS.

## Introduction

Potocki-Lupski syndrome (PTLS) is a very rare chromosomal anomaly (1:25.000 people worldwide), resulting from the partial duplication of the short arm of chromosome 17 (17p11.2 microduplication) (1). PTLS was first reported in 2007, although the first case described appears to be from 1996. The name of the disease is associated with the two researchers who described it, Lorraine Potocki and James R. Lupski (2). PTLS is a developmental disorder with features ranging from mild to severe ones. It is characterized by hypotonia, developmental and intellectual delay, or congenital anomalies. The variability of the clinical presentations is more important regarding the cognitive level and behavioral disorders (3).

Children with PTLS have a characteristic phenotype with facial dysmorphism and neurological or behavioral abnormalities. The craniofacial dysmorphic features include the triangular or oval face, micrognathia (in early childhood), high-arched palate, down slanting palpebral fissures, broad forehead, protruding nose, smooth chin, and dental malocclusion (1, 4). The neurological manifestations are variable and give the prognosis of the disease. These children may present speech and language impairment, delayed developmental milestones, intellectual disability, behavioral disorders (repetitive behaviors, anxiety, withdrawal, attention deficit hyperactivity disorder—ADHD, autism spectrum disorders—ASD), motor clumsiness/coordination impairment, or other neuropsychiatric disorders (1, 3, 5). The natural course of PTLS involves hypotonia, feeding difficulties in infants, and sleep disturbance (obstructive or central sleep apnea), more evident as the child gets older, without common anomalies revealed on brain MRI (1, 3, 4).

In most cases, the genetic abnormality is *de novo*, and less often, the transmission is autosomal dominant (1). There are few genes involved in PTLS etiopathogenesis, RAI1, SREBF1, DRG2, LLGL1, SHMT1, or ZFP179 (6). The diagnosis of PTLS is established by detecting duplication that encompasses the RAI1 (retinoic acid 1) gene at chromosome 17p11.2. This duplicated region may contain several genes, but RAI1 underlies the main features of PTLS. The recurrent duplication accounts for approximately two-thirds of 17p11.2 duplications and non-recurrent duplications for about one-third of cases. An additional copy of the RAI1 gene underlies many of the characteristic features of PTLS (7). Also, RAI1 controls the expression of genes involved in the sleep-wake cycle, thus explaining sleep disorders in children with PTLS. But the involvement of the RAI1 gene in triggering intellectual disabilities and neurological manifestations has not been proven. Most likely, they are the result of duplication of the other genes involved.

The exact incidence of these disorders is unknown. More than 50 children with PTLS have been described in the literature so far (1).

## Case Presentation

**Figure 1:**
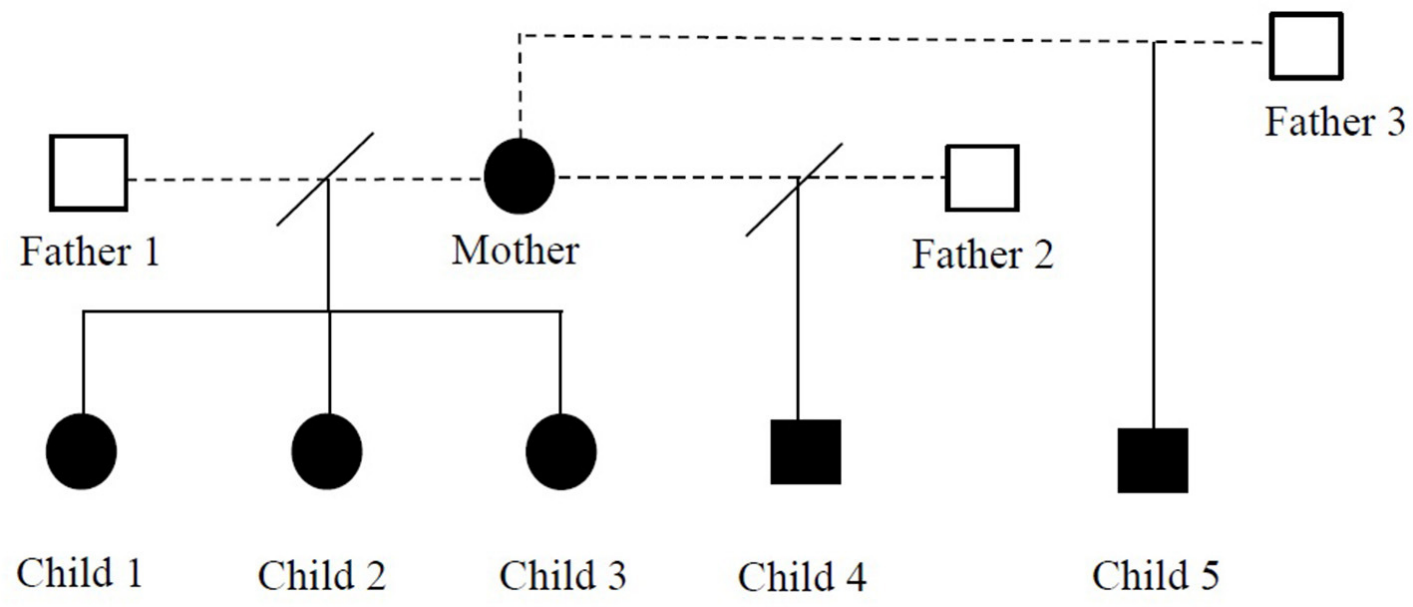
Pedigree chart of the reported family with Potocki-Lupski syndrome.

We report a family (five children and a mother) with 17p11.2 microduplication. As far as we know, this is the first described family with PTLS in Romania. The children have three different fathers. The written informed consent was obtained from the children's mother (as sole legal guardian) to publish the cases and any accompanying images. The pedigree analysis of this family is presented in Figure 1.

**Figure 2:**
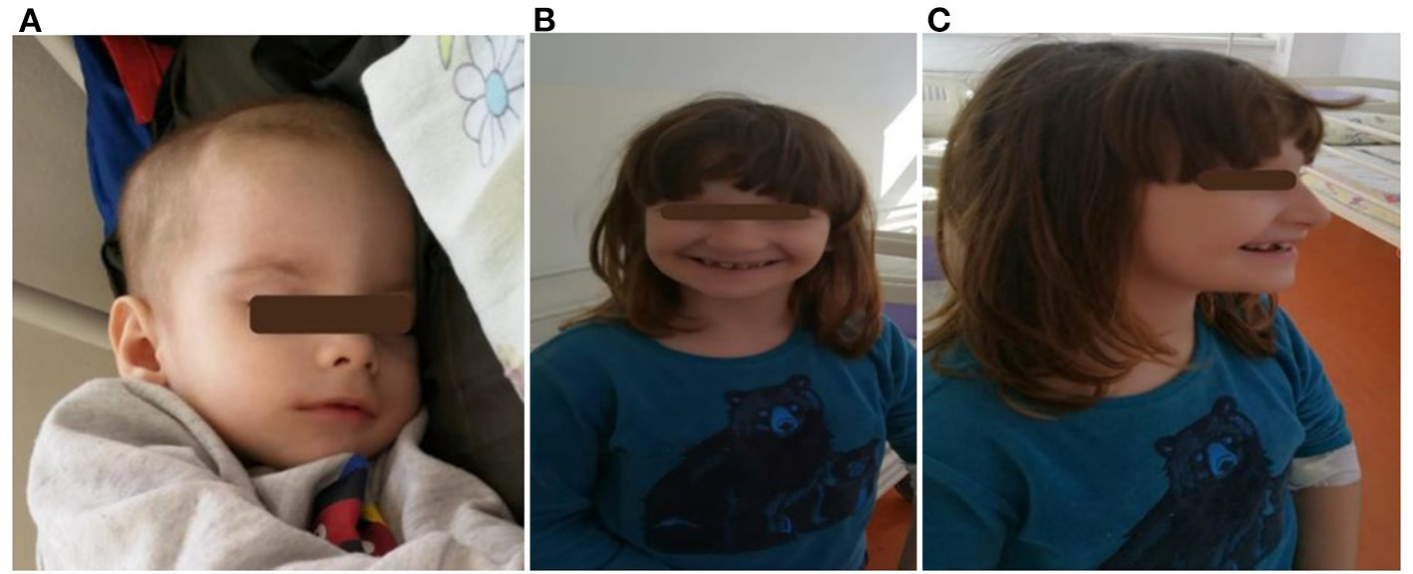
Two siblings: **(A)** a 3 month-old infant (child 5) with craniofacial dysmorphism; **(B,C)** a 5- year 5-month-old girl (child 3) with craniofacial dysmorphism, mandibular hypoplasia, dental abnormalities, malocclusion, and intellectual disability.

The diagnostic was established when the fourth brother (child 4) was referred to us at the age of 2 years and 10 months for an acute respiratory infection. He was born at 37th week of gestation from an unmonitored pregnancy, vaginal delivery. The mother was 33 years old, from a poor background, and with a liminal intellect. None of the pregnancies have been monitored, but the mother denied infections or other problems. At first admission in our unit, the child's physical examination revealed facial dysmorphic features, neurological disorder, and severe malnutrition due to feeding difficulties. The boy's weight was 7.3 kg (−3.7 SD), height 75 cm (−3.75 SD), and head circumference 42 cm (−2.7 SD). His facial dysmorphism was impressive: triangular face, micrognathia, dolichocephaly, long chin, down slanting palpebral fissures, and bulbous nose tip. His ears were very prominent, which gave him the appearance of an elf (Figure 2). The neurological examination showed mild axillary hypotonia, speech and language impairment, and developmental delay (Table 1). We performed genetic testing, including the karyotype and multiplex ligation-dependent probe amplification technique (MLPA) for the main microdeleted/microduplicated regions (using SALSA MLPA Probe mix P245 Microdeletion Microduplication Syndromes kit). MLPA test showed the duplication of three regions (RAI1, DRC3-6, LLGL1-4RA) of 17p11.2 chromosomes corresponding to PTLS.

**Table 1:** Clinical and genetic data of the reported family compared to reported features in children with Potocki-Lupski syndrome (1–10).

**Clinical and genetic data**	**Child 1**	**Child 2**	**Child 3**	**Child 4**	**Child 5**	**Mother**
Age at diagnosis	13 yrs	8 yrs, 2 mos	5 yrs, 5 mos	2 yrs, 10 mos	3 mos	33 yrs
Sex	Female	Female	Female	Male	Male	Female
Genetic test (MLPA)	17p11.2 microduplication (RAI1, DRC3-6, LLGL1-4)					
Malnutrition BMI z-score	No 0.2 SD	Yes −2.4 SD	Yes −2.8 SD	Yes −3.7 SD	No 0.12 SD	No 0.5 SD
Facial features						
Micrognathia Down-slanting palpebral fissures Broad forehead Long nasal tip Triangular face The prominence of the angle of the jaw Oval-shape face and longer chin Microcephalia Hypertelorism Low-set and posteriorly rotated ears Asymmetric smile	+ - - + - - + - + + +	+ - + + + - + + + + +	+ + + + + - + + + + +/-	+ + + + + + + + + + -	+ + + + + + + + + - -	+ - - + - - + - + - +
Neurodevelopmental features						
Mild-to-moderate infantile hypotonia Oropharyngeal dysphagia Poor feeding Mild-to-moderate gross motor delays	- - - -	- - - +	- - + +	+ + + +	+ + + -	- - - -
Cognitive impairment Developmental milestones delay peech delay (expressive and receptive language impairment, articulation difficulties, disordered intonation, prosody) Verbal apraxia	+ - + -	+ - + -	+ - + -	+ - + -	- - - -	+ - + -
Sleep-disordered breathing (mild central and/or obstructive sleep apnea)	-	-	-	-	-	-
Behavioral difficulties (attention deficit, withdrawal, hyperactivity, anxiety, ADHD)	+	-	-	-	-	-
Autism spectrum disorders (ASD) Autistic features (decreased eye contact, motor mannerisms, posturing, sensory hypersensitivity, repetitive behaviors, difficulties with transitions, lack of appropriate functional or symbolic play, lack of joint attention)	- -	- -	- -	- -	- -	- -
Congenital heart disease						
Atrial septal defect Ventricular septal defect Bicuspid aortic valve Dilated aortic root Hypoplastic left heart Arrhythmia	- - - - - -	- - - - - -	- - - - - -	- - - - - -	- - - - - -	- - - - - -
Growth hormone deficiency						
Short stature Hypoglycemia	- -	- -	+ -	+ +	- -	- -
Musculoskeletal features						
Severe bilateral clubfoot Joint hypermobility Kyphoscoliosis Flat foot Long fingers and toes	- - - - -	- - - - -	- - - - -	- - - - -	- - - - -	- - - - -
Renal anomalies						
Hypoplastic kidneys Multi-cystic dysplastic kidneys Hydronephrosis	- - -	- - -	- - -	- - -	- - -	- - -
Others						
Hyperopia Mild high-frequency sensorial hearing loss Dental malocclusion or dental crowding	- - +	- - +	- - +	- - -	- - -	- - +

*+, present; -, not detected; yrs, years; mos, months; SD, standard deviation; MLPA, multiplex ligation dependent probe amplification; BMI, body mass index; RAI1, retinoic acid induced 1; DRC, dynein regulatory complex; LLGL, lethal giant larvae homolog*.

**Figure 3:**
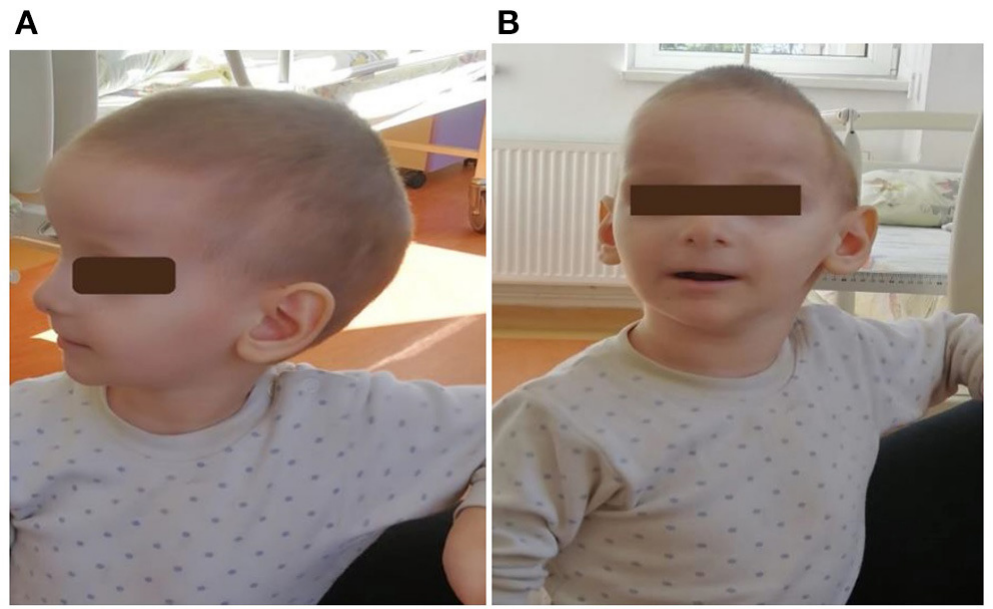
**(A,B)**: First patient (child 4) at 2 years and 10 months. He had craniofacial dysmorphism, ears with thin helices, deep conchae, and hypoplastic lobules.

Subsequently, the other four siblings (an older sister 13 years old, a girl 8 years 2 months old, the youngest girl, 5 years 5 months old, and the smallest brother, aged 3 months) were evaluated. There were no records of the medical history or developmental milestones for the children of this family as they were not registered to a family physician. All children had the same typical disease manifestations (facial dysmorphism, dolichocephaly, prominent ears, and neurologic disorder (Figure 3; Table 1). Also, feeding difficulties and axial hypotonia were observed, especially in the two younger brothers. No sleep disorders or behavioral problems were reported in these children, other than ADHD in the older one. On their first clinical examination, three siblings were underweight, and one child also had short stature (z-score −2.17 SD). All brothers were genetically tested by MLPA analysis, and all of them presented 17p11.2 microduplication (RAI1, DRC3-6, LLGL1-4RA). The duplication was also detected in the peripheral blood of their mother, suggesting the autosomal dominant transmission. The cardiology exam (including electrocardiogram and echocardiography) revealed no pathological changes in all children. Also, abdominal ultrasound examination (including renal examination) and laboratory parameters (electrolytes, liver or renal function tests, and bone metabolism) were in normal ranges.

Even though they have a mild form of PTLS, presenting just some of the clinical features described in this disease, the children are followed now by a multi-disciplinary team including pediatricians, a nutritionist, a neurologist, a psychiatrist, a psychologist, speech therapy specialists, and social-aid workers. We aim to improve their prognostic for the long-term and to minimize intellectual and behavioral problems.

## Discussions

We presented a mother and her five children (three girls and two boys) diagnosed with PTLS (17p11.2 microdeletion). All of them had the main clinical manifestations of the disease, respectively craniofacial dysmorphism and neurological impairment. Fortunately, none of them had heart damage, kidney or osteoarticular anomalies, or sleep disorders, often described in patients with the disease (Table 1). Only one child was diagnosed with ADHD, but the others report no behavioral problems.

Duplication 17p11.2 syndrome is also frequently associated with failure to thrive, as seen in our patients (three of the five children had a growth deficit at their first assessment). Malnutrition may be due to gastroesophageal reflux or swallowing difficulties (dysphagia), leading to feeding problems during infancy. In one of our children, the height was also reduced. Short stature may be due to severe malnutrition or growth hormone deficiency when it is often associated with hypoglycemia (1, 2, 4, 8, 9).

Congenital malformations may be present in children with PTLS. Cardiovascular anomalies (dilated aortic root, bi-commissural aortic valve, atrial/ventricular, and septal defects) are described in ~40% of children with PTLS. The connective tissue damage found in children with PTLS explains a more frequent aorta or aortic valve involvement, the most common abnormality being aortic root dilation (8). Congenital heart defects can be severe and progress to severe heart failure, requiring heart transplantation. In 2011, the first case with PTLS with heart transplantation had been reported: a 10-year-old boy with 17p11.2 duplication and hypoplastic left heart syndrome (9). Genetic factors determine the cardiovascular damage in PTLS. Our children had no signs of structural heart disease, aortic or valvular disorders, but they need periodic cardiovascular monitoring given the risk for cardiovascular involvement. Kidney anomalies have been observed in over 10% of affected individuals. The most common are hypoplastic kidneys, hydronephrosis, or multi-cystic dysplastic kidneys. Ocular, musculoskeletal, and osteoarticular abnormalities are also described but less frequently (4, 6–9, 11).

PTLS may be suspect based on the child's appearance. The phenotype is often not so suggestive for this disease. Genetic testing must be used in a patient with an intellectual disability or other psychiatric or neurologic signs associated with facial dysmorphism, congenital anomalies and/or failure to thrive. In 2013, the first case of PTLS with the prenatal diagnosis was described. It was a fetus with hypoplastic left heart and aberrant right subclavian artery in which fetal karyotype was performed by amniocentesis (10).

Differential diagnoses of PTLS include Smith-Magenis syndrome (SMS), Down syndrome, Williams syndrome, brachydactyly-intellectual deficit syndrome (del 2q37), Prader-Willi syndrome, or Sotos syndrome. The first condition that needs to be excluded is SMS, a rare syndrome with similar clinical manifestations and chromosome 17 involvement (deletion or a mutation in the RAI1 gene) (12). Both PTLS and SMS occur because of a non-allelic homologous recombination defect involving a 1.3–3.7 Mb of 17p11.2 chromosomal region in which the RAI1 gene is found. Other genes that have been identified in this region are SREBF1, DRG2, LLGL1, SHMT1, and ZFP179 (12–14).

There is no specific treatment for children with PTLS. Each child will benefit from an individualized treatment adapted to his needs, including speech therapy, physical therapy, behavior and communication therapies, or different specialized education services. Managing children with severe forms (congenital heart defects, renal or gastrointestinal disorders) requires a multi-disciplinary team. Collaboration between specialists in pediatrics, cardiology, genetic disease, neurology, psychiatry, psychology, nutrition, nephrology, and orthopedics is mandatory in caring for these children (13, 14). Genetic advice is essential in such cases. After the first four children's diagnosis, the mother (who was already pregnant with the 5th child) was informed about the risk of the disease in subsequent pregnancies. With this last pregnancy, she decided to ligate the fallopian tubes.

A limitation in the genetic analysis was the impossibility of the precise delimitation of the region involved in duplication to indicate if it is a recurrent or a non-recurrent one and, therefore, suggesting some correlations between an accurate copy number variant and the phenotype. Also, it was not possible to perform genetic testing by chromosomal microarray for these patients. However, we considered it necessary to report this family with an affected mother, diagnosed only after the birth of all her five affected children. Also, the lack of specificity of the clinical presentation in PTLS indicates the need for a more rigorous evaluation of individuals with intellectual disability (isolated or syndromic) by genetic testing, as chromosomal microarray or MLPA technique (13). An accurate diagnosis of the mother before pregnancies would have been beneficial for providing timely genetic counseling, including the high risk of recurrence (50%), thus helping the mother and her family make an informed decision regarding future pregnancies.

## Conclusions

Children with PTLS are at a high risk of neurodevelopmental disorders (especially learning and language disabilities) or ASD, leading to behavior problems or social interaction and communication difficulties. In a child with suggestive phenotypes for a genetic disease, the main aim of the pediatrician is an early diagnosis to prevent possible complications. A multi-disciplinary team is needed for these cases. The parents of these children should be counseled and helped to deal with medical problems, psychological behavior, and neuropsychiatric disorders that may occur because the prognosis of these children is difficult to predict. Moreover, extremely important is genetic advice.

## Data Availability Statement

The original contributions presented in the study are included in the article, further inquiries can be directed to the corresponding authors.

## Ethics Statement

Written informed consent has been obtained from the parents of the patients to publish this paper. Written informed consent to participate in this study was provided by the participants' legal guardian/next of kin. Written informed consent was obtained from the minors' sole legal guardian (mother) for the publication of any potentially identifiable images or data included in this article.

## Author Contributions

AG, DM, and TLP: methodology. AG, CS, DM, SSC, BB, DH, and TLP: formal analysis. AG, CS, and TLP: writing—original draft preparation. AG, CS, DM, and TLP: writing—review and editing. All authors have read and agreed to the published version of the manuscript.

## Conflict of Interest

The authors declare that the research was conducted in the absence of any commercial or financial relationships that could be construed as a potential conflict of interest.

## Publisher's Note

All claims expressed in this article are solely those of the authors and do not necessarily represent those of their affiliated organizations, or those of the publisher, the editors and the reviewers. Any product that may be evaluated in this article, or claim that may be made by its manufacturer, is not guaranteed or endorsed by the publisher.
